# Rayyan.ai

**DOI:** 10.29173/jchla29964

**Published:** 2026-08-03

**Authors:** Vanessa Kitchin

**Affiliations:** Knowledge Synthesis & Medical Librarian, University of British Columbia, Vancouver, BC, Canada

**Product:** Rayyan.ai

**URL:** https://www.rayyan.ai/

## Purpose

Rayyan.ai is a web-based platform designed to support workflows in evidence syntheses. It has an application available both for iOS and Android that is designed for mobile screening. Rayyan was originally released as a tool to support title and abstract screening but has expanded into AI-assisted decision-making and data extraction. This shift reflects a growing movement toward automation in evidence synthesis practices [1] but also raises important methodological concerns regarding transparency and reproducibility, not to mention acceptability for the purpose of publication.

## Product description

Rayyan.ai allows users to import citations, deduplicate records, and conduct title and abstract and full-text screening in a collaborative environment. Reviews can be blinded (each reviewer works independently) or unblinded (all reviewers can see each other’s decisions) and users can apply labels, filters, notes, and conflict resolution tips within the platform to aid decision making.

When first using the tool, it quickly becomes apparent that many of its features are restricted behind a paywall. In fact, this limitation prompted me to contact the platform to request a free trial to support the completion of this product review. The team was highly responsive and kindly provided a 14 day trial period. I was also offered the opportunity to meet with a Support Manager, Bashar Bosheh, who provided a live demonstration and tour of the platform.

Bashar explained that Rayyan.ai’s decision-making is based on a traditional machine learning classifier rather than a large language model (LLM) such as ChatGPT. According to Rayyan’s help documentation [2], their “prediction classifier” uses a Support Vector Machine (SVM) that is trained on features extracted from article titles and abstracts. As reviewers manually screen studies and make inclusion or exclusion decisions, the system learns from these choices. Once a sufficient number of decisions have been made (Rayyan indicates at least 50 total decisions, including a minimum of five inclusions and five exclusions), the model begins generating predictions for unscreened records, suggesting whether they are likely to be included or excluded. This means the tool relies on pattern recognition to support screening decisions.

Bashar demonstrated how to use and customize the various AI tools within the platform, and also showed how to upload full-text PDFs. When asked about licensing considerations and the handling of non-open access articles, he explained that Rayyan.ai does not verify the copyright or licensing status of each uploaded paper. Responsibility for ensuring PDFs are used in accordance with publisher agreements, copyright law, and institutional subscription terms remains with the user. This is important because access to an article through a university library subscription does not necessarily grant permission to redistribute that PDF through a third-party platform. In practice, users should consider whether the article is open access, whether uploading creates a stored copy on external servers, and whether publisher terms restrict sharing. Bashar further noted that if the AI features are used to support a review intended for publication, it is essential to check the specific policies of individual journals and publishers regarding AI use. He also acknowledged that many journals currently do not permit or accept the use of such tools.

### AI Features

Rayyan has the following AI tools:

- ResearchPilot
  An AI assistant that allows users to query the imported citation set, identify potentially relevant studies, and prioritize records. While useful, it may create bias where early reviewer decisions influence later decisions.
- AI Reviewer
  Applies user-defined inclusion and exclusion criteria to records and generates recommendations with rationale.
- AI Analyzer
  Provides a summary of how a record aligns with screening criteria but does not make a final inclusion/exclusion decision as that still remains the responsibility of the human reviewer. Rather than replacing reviewer judgment, it acts as a decision-support feature.
- AI-Assisted Data Extraction
  Users can build custom extraction forms, and the platform highlights and extracts relevant text that it believes corresponds to user-generated fields.

**Fig. 1:**
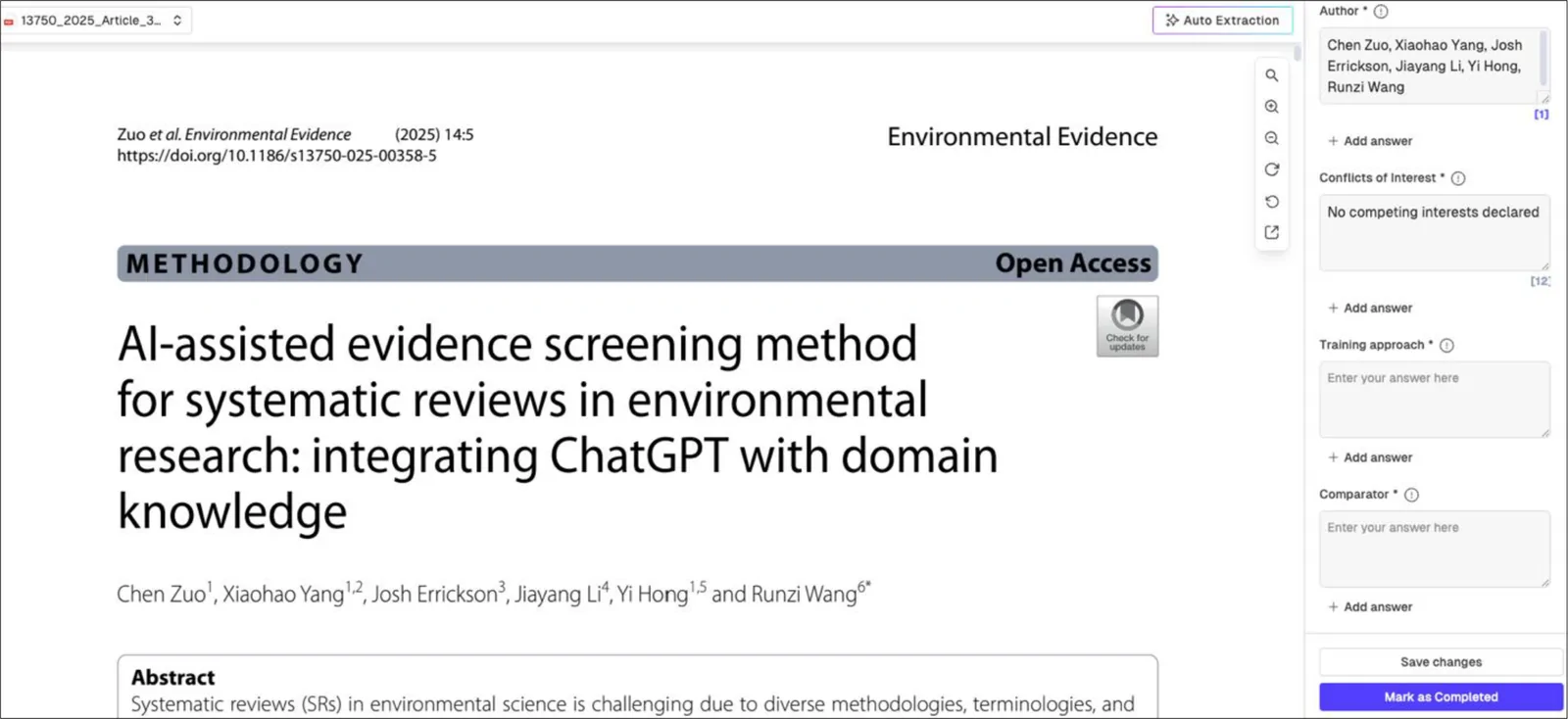
AI-assisted data extraction form in Rayyan.ai

For my test review, I examined papers assessing the effectiveness of AI-based screening tools in systematic and scoping reviews. My data extraction fields included items such as author details, conflicts of interest, the AI training approach, the type of AI used, the comparator, and other variables. In my testing, AI-assisted data extraction populated the requested fields only about 20% of the time. Even when fields were completed, the outputs were inconsistent and required manual verification. The remaining fields were frequently left blank, resulting in incomplete extraction and the need for substantial follow-up. I found that anything beyond basic bibliographic or declarative fields (like authorship and conflicts of interest) was frequently left unfilled (Figure 1). This may be less prominent for more quantitative extraction fields with defined variables, but it raises important limitations around reliability and completeness of an AI data extractor. Based on my experience, I would be cautious about relying on this tool as a fully automated or “blind” data extractor without substantial human verification.

## Intended users

- Researchers conducting systematic, scoping, or related reviews
- Librarians supporting evidence syntheses
- Students and trainees learning review methods
- Small teams seeking collaborative screening software

## Special features

- Blinded screening for independent reviewers
- Tagging, filtering, and notes
- Conflict resolution tools
- AI Reviewer and AI Analyzer
- ResearchPilot (AI assistant)
- Custom data extraction forms
- Mobile application for screening
- Duplicate resolution in premium version

## Compatibility and workflow issues

Rayyan.ai accepts common citation export formats such as RIS, XML, CSV, PDF and others, and therefore integrates well with most database outputs. Deduplication requires premium access so users may need to manually manage duplicates in an external tool such as EndNote or Zotero before importing if they are unable to upgrade.

## Platform

Rayyan.ai is browser-based and works across all operating systems. A mobile app is available, though advanced functions are more practical on desktop.

## Usability

Rayyan.ai’s strongest feature remains its screening interface. It is clean, intuitive, and efficient for title and abstract review.

The AI features are weaker. AI Reviewer outputs can become time-consuming when they do not function reliably. As such, the workload may shift from initial screening to manual verification, correction of inaccurate suggestions, and completion of missing data extraction fields. This potentially increases rather than reduces reviewer effort. Similarly, if AI-generated decisions are mixed with human decisions, conflict resolution may become more complicated.

## Strengths

**Fig. 2:**
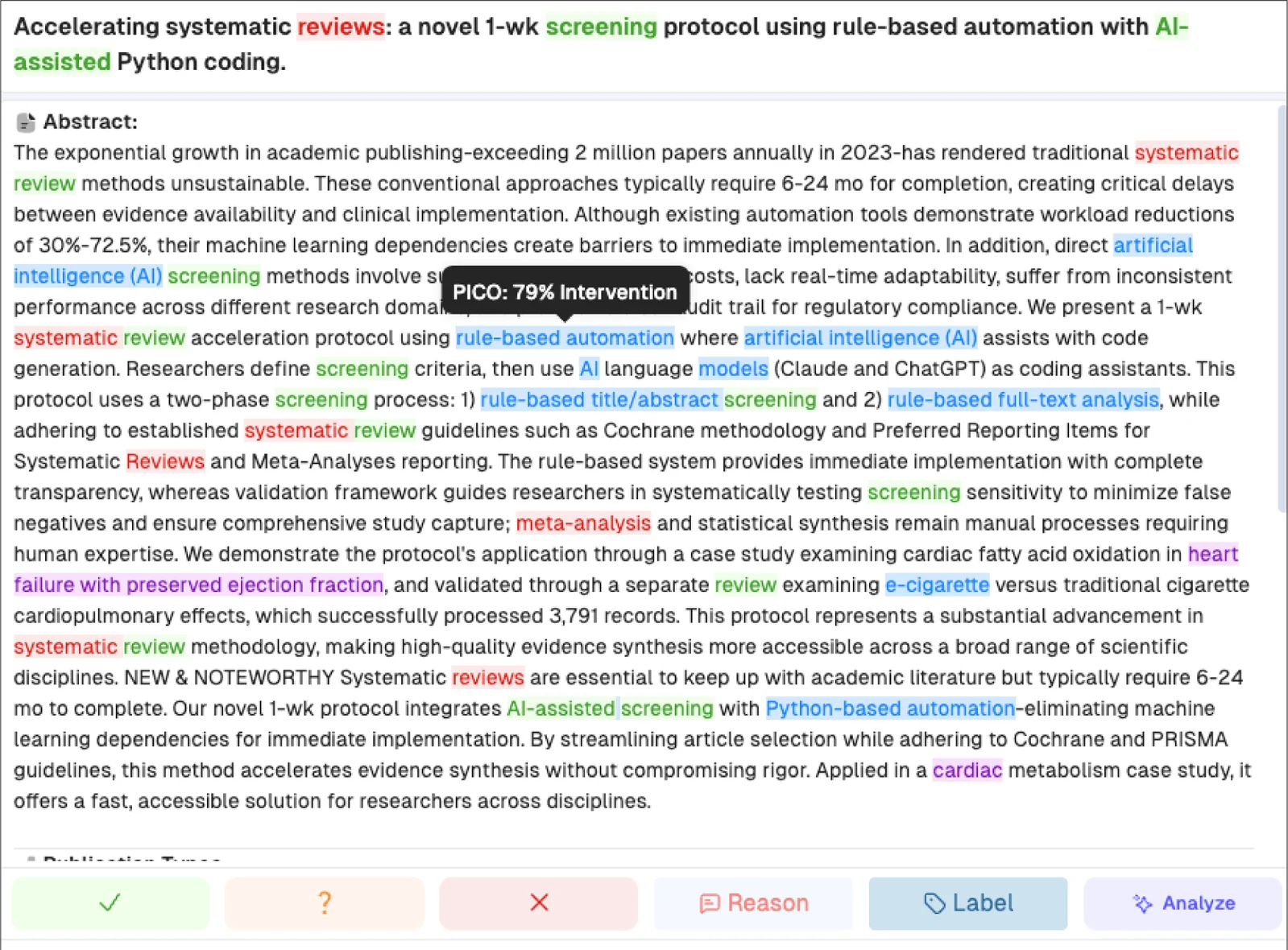
PICO Highlighting & Filtering tool in the Screening phase

- User-friendly screening interface
- Excellent collaborative screening features
- Free version lowers access barriers
- Useful tagging and filtering tools. These include decision status filters; reviewer filters (shows records screened by a specific reviewer); label filters based on custom tags; keyword filters; and finally, exclusion reason filters. Further to these, Rayyan.ai also offers PICO highlighting which is an AI-assisted filter that highlights all the PICO related terms and phrases and then extracts them to three “filtration facets”: Population, Intervention/Comparison, and Outcome. The PICO terms are pulled automatically from the research question and you can customize those as necessary. Rayyan.ai uses distinct colours to highlight terms based on the PICO element they belong to (Figure 2). When the user hovers over the highlighted terms, the percentage of probable relevancy is also indicated.
- Active product development and support availability
- AI tools may help with exploratory or rapid reviews

## Weaknesses

- Core workflow features limited by paywalls
- AI decision-making lacks full transparency
- AI outputs require complete human verification
- Potential bias from prioritization algorithms
- AI tools add complexity during conflict resolution
- Data extraction is inconsistent and unreliable
- Can jeopardize journal acceptance when using AI-assisted methods

## Comparison with similar products

Compared with Covidence, Rayyan.ai offers more AI-integration and a partially free tier. However, Covidence provides a more structured workflow, especially for deduplication, extraction, and overall review management. I personally prefer the Covidence user interface although Rayyan.ai’s is also strong.

**Fig. 3:**
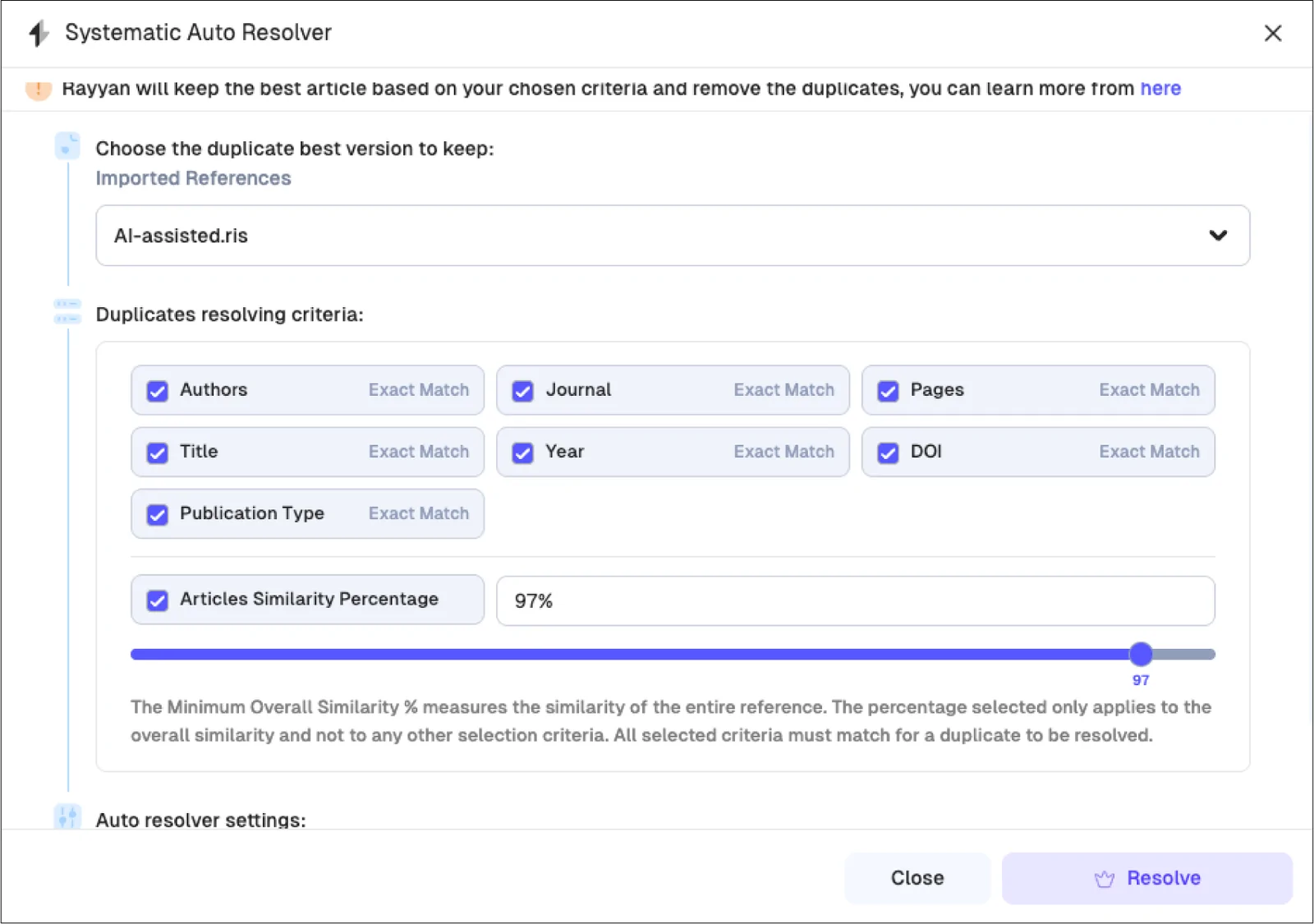
Rayyan.ai’s deduplication options

That said, Rayyan.ai offers more opportunities for customization. For example, deduplication can be fully customized as shown in Figure 3.

**Fig. 4:**
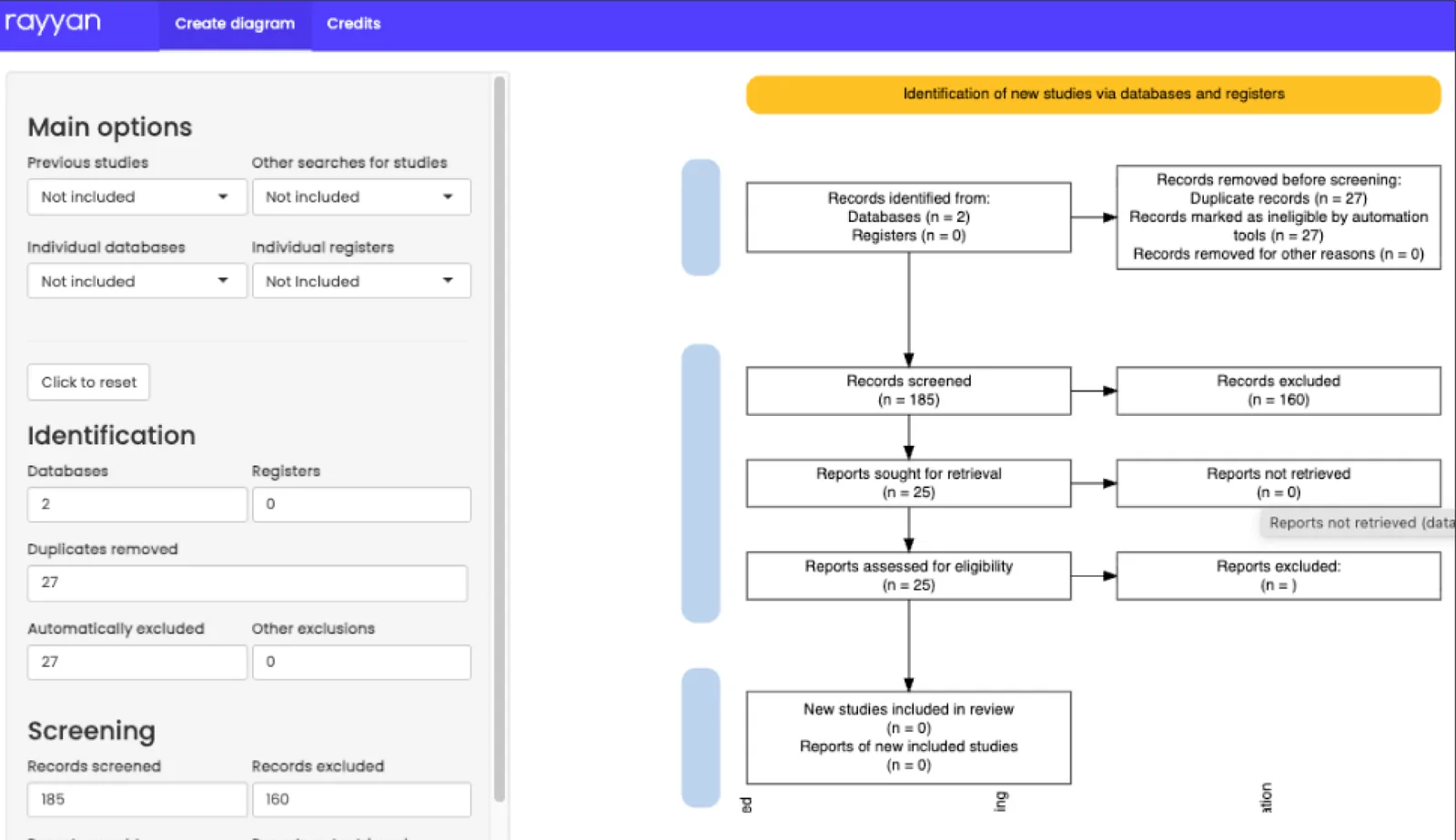
PRISMA diagram generation on the Rayyan.ai platform

Further, Rayyan.ai’s PRISMA diagram generation is more customizable and truer to the PRISMA diagram format (Figure 4).

## Cost/value

While Rayyan does offer a free version with screening capability, the paid tier unlocks AI tools and robust deduplication as well as enhanced workflow functions. For students and smaller teams learning about methodology, the free version may provide value; however, for publication-focused reviews, premium pricing should be weighed against alternatives like Covidence.

## Conclusion

AI tools have not yet clearly demonstrated equivalence to fully human PRISMA-aligned workflows. Concerns include:

- Opaque or “black box” screening decisions • Reduced reliability in data extraction
- Limited reproducibility
- Need for full human oversight

Recent evaluations [3,4] further suggest that while AI-assisted screening tools may show promising sensitivity, they often demonstrate lower specificity and variable performance when applied to tasks such as data extraction. Rayyan.ai is an accessible platform for screening studies. The platform’s newer AI-enabled features can enhance workflow efficiency, particularly for prioritizing records in large datasets or supporting rapid or exploratory reviews. However, for evidence syntheses intended for publication that require strict adherence to methodological standards, these AI features should be used cautiously. At present, they are best understood as productivity aids that can streamline parts of the screening process, rather than replacements for independent human reviewers.
